# Transferring knowledge into practice? Exploring the feasibility of action learning for improving knowledge, skills and confidence in clinical communication skills

**DOI:** 10.1186/s12909-019-1467-4

**Published:** 2019-01-28

**Authors:** Jane Dowson

**Affiliations:** University of Liverpool, Liverpool, UK

**Keywords:** Knowledge transfer, Action learning, Reflexivity, Coaching, Professional practice, Behavioural change, Organisational learning, Education, Training, Skills

## Abstract

**Background:**

Effective communication between patients and practitioners is fundamental to the delivery of high-quality care. This is particularly important in the complex and challenging nature of working in palliative and end of life care. Following specialist communication skills training, a group of healthcare professionals explored the impact of action learning (AL) on the perceptions of their knowledge, skills and confidence in communication skills. The research also aimed to establish an evidence base by exploring the nature and impact of the AL approach employed to facilitate improvements in professional practice.

**Methods:**

The research employed a mixed methods approach. Learners (*n* = 57) scored their perceptions in key areas of communication skills through questionnaires as a baseline measure at time point T0. From this group, 12 participants were selected to undertake further follow-up, divided into Control (*n* = 6) and Intervention arms (n = 6). All repeated the same questionnaire at 3 subsequent time points (T1, T2, T3) scheduled monthly. Half also received additional telephone-coaching conversation intervention based on Weber’s TLA® critical and reflexive approach (2014). To explore and assess perceptions, similarities and differences of their experience of undertaking the specific AL approach and processes, all completed participants (*n* = 4) and coaches (*n* = 2) were interviewed at time point T4 (33% response rate). Quantitative data from questionnaires was analysed for comparison of variables to provide a visual illustration of perceived learning journeys. Qualitative data from coaching conversations, interviews and questionnaire responses was analysed inductively to create final themes.

**Results:**

Perceived improvements in knowledge, skills and confidence measured at 35% at time point T0, and improvement of 46% reported at time point T3. In the Control arm this was calculated at 25%, and at 67% from the Intervention arm. Findings indicate encouraging evidence for perceived improvements of knowledge, skills and confidence.

**Conclusions:**

The research demonstrates a positive appetite for, and experience of, the process and method. The value of such a solution-focused, critically reflexive AL practice suggests this may act as a facilitator for successful transfer of learning into practice for individuals and their organisations.

## Background

### Context and purpose of study

Effective communication between patients and practitioners is fundamental to the delivery of high-quality care and practitioners’ communication skills can be improved through experiential learning [1]. Research evidence shows that effective communication between clinicians and patients is not always an automatic by-product of clinical experience [2] therefore effective learning transfer into practice is particularly important in the complex and challenging nature of working in palliative and end of life care. Many training and development programmes within healthcare have embedded the process of including action into curricula, drawing upon important works [3–9] to foster motivational intrinsic behaviours to enable practical application after training is completed. However, the close relationship between action and learning which became systematised through important works, such as Revans’ [10–12] saw a shift in focus in the methodology and process of action learning, using change as an iterative process to help solve complex, critical, and urgent problems [13, 14]. Successful and effective learning transfer can be guided by a shift in the behaviours that course participants focus on at the end of skills training. A reflexive and action learning approach can facilitate not only short term skills improvement but extends learning towards change management [15, 16]. Therefore the research was designed as an initial study in 2016/2017 to explore the nature and impact of action learning with a group of healthcare professionals following their training in specialist communication skills for Palliative and End of Life Care.

### Aims & objectives

In order to understand the extent to which AL might potentially support and facilitate the transfer of learning into practice, the research aimed to:

- Explore the nature and impact of AL on the perceived levels of knowledge, skills and confidence of learners over time, and
- Establish feasibility and an evidence base for adopting this type of critically reflexive AL approach

By measuring any changes in the reported levels of knowledge, skills and confidence over time, and exploring any differences in those perceptions, the research was able to assess how, and to what extent, the specific AL approach may facilitate improvements to professional practice for this group of healthcare professionals. Additionally, learners’ experiences and perceptions of this approach may indicate a level of utility for further development or refinement of future education and training provision in this area.

## Methods

### Study design and population

The research employed a mixed methods approach, designed as a sequential and longitudinal study. Courses in communication skills training for Palliative and End of Life Care healthcare professionals are provided regularly throughout the northwest region of the UK and questionnaires are frequently used as a self-assessment tool for course participants themselves as well as course providers in evaluating course provision. However, to date there has been little or no published research exploring Weber’s (2014) TLA® intervention within medical and healthcare education. Therefore, this provided an ideal population to explore its potential impact. The methodologies employed in this research study were designed to increase the researchers understanding of the perceived levels of knowledge, skills and confidence that may occur at different time points, and how a specific action learning intervention may facilitate learning transfer into practice.

**Fig. 1:**
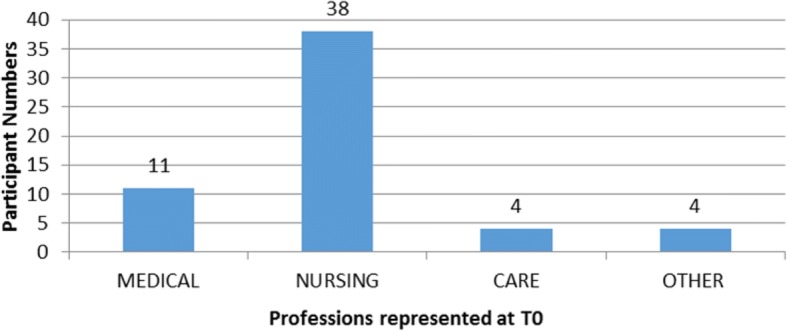
Participants professions at the time of Communication Skills training provision

Fifty seven participants from a variety of disciplines and healthcare settings undertook training in Advanced (ACST), Intermediate (ICST) or Core (CCST) Communication Skills, within the North West of England, between January and July 2016, as illustrated in Fig. 1. Participants attended the level of training based on their individual and organisational learning requirements, supported by the Cheshire & Merseyside Palliative and End of Life Care clinical network (C&MPEoLCCN) who co-ordinated training. Nurses were the only profession to attend all three types of training course provided. Medically qualified healthcare professionals attended only ACST training with health and social care professionals attending Core levels of training. Females represented over 90% of the participants and over half (54%) of the participants worked at healthcare organisations within the Liverpool city region.

### Description of processes, interventions and comparisons

**Fig. 2:**
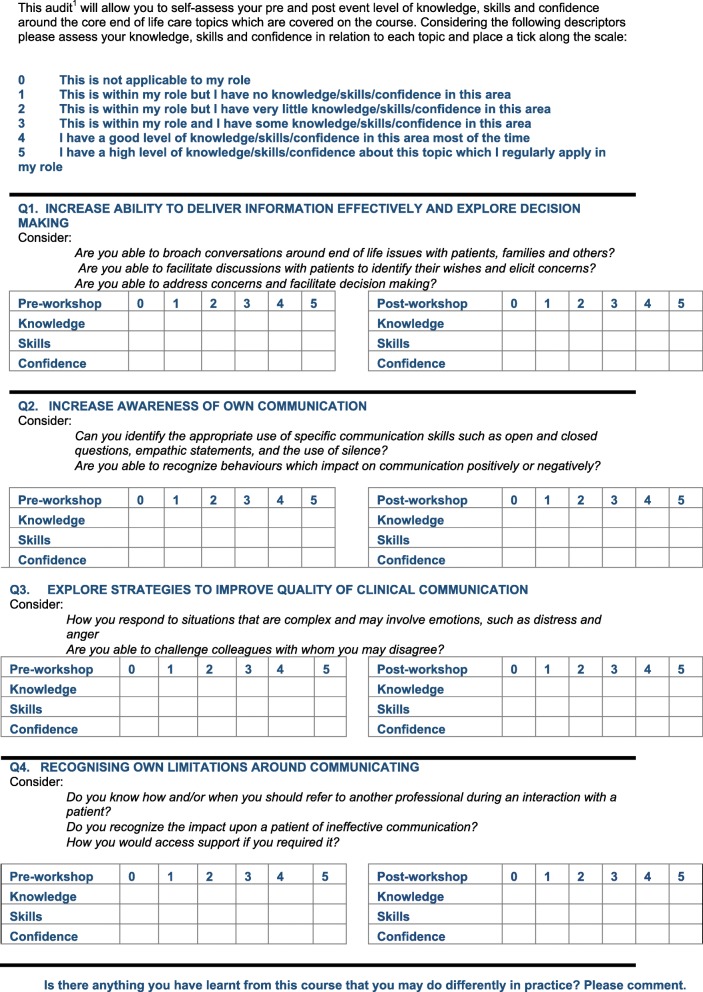
Sample questionnaire for ACST (adapted)

All 57 participants undertook a pre-validated questionnaire at time point T0, prior to course commencement (pre) and immediately on course conclusion (post). This measured their perception of their level of knowledge, skills, and confidence (KSC) in undertaking clinical care following their attendance and participation in the Communication Skills training course (Fig. 2). At the close of each course that took place between January and July 2016, the research study was presented to course participants. All participants provided written consent for their data to be used for the dual purpose of providing the researcher with a baseline measure at T0, as well as for standard course evaluation purposes. For the follow-up aspects of the study (T1–4), 12 healthcare professionals were selected from the group of 57. The study received full ethical approval and additionally R&D approval from relevant NHS Trusts before the first course was undertaken in January 2016.

### Study measures

The questionnaire shown below is an example of the questionnaire used for ACST course participants (the majority of participants), and shown for time point T0. The same questionnaire was used throughout all time points, and was adapted for participants undertaking ICST and CCST. It can be seen that although the main assessment mechanism utilises a self-assessment scoring approach, there is space for free text ability and this was encouraged at all time points.

**Fig. 3:**
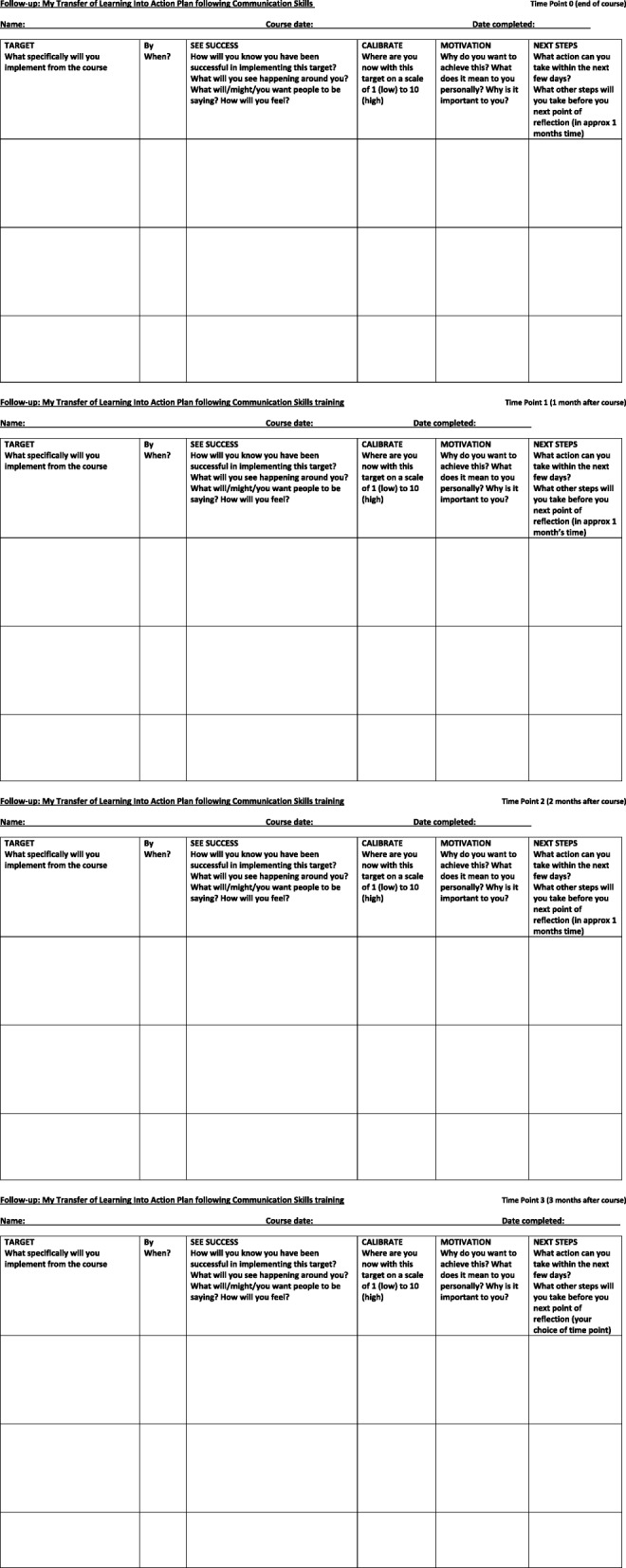
Example of Action plan and recording template

To compare any differences in employing Weber’s (2014) TLA® AL approach, the 12 research participants were divided randomly into two study arms, Control, and Intervention. All participants completed repeat post-course questionnaires at scheduled monthly time points (T1-T3) after their training in Communication Skills at T0. Intervention arm participants received additional telephone-based coaching [15, 16] at the same time points. To assist with reflexive learning, all participants were provided with an action planning template [15, 16] (Fig. 3) and if they so wished, participants could choose to use this for reflexive purposes when completing questionnaires and/or preparation for telephone coaching discussions.

Triangulation through open ended, semi-structured interviews [17] at the end of the action learning process (T4) was employed to obtain completed participants’ perspectives on their experience of the process. This included exploration of any potential facilitators and barriers to the use and feasibility of follow-up action learning processes for future participants following training provision. Additionally, the perspectives of the coaches providing the intervention was also sought via semi-structured interviews, to elicit their thoughts around the process and potential feasibility and/or further development of the concept for future use. It was important to explore the experience of coaches, particularly as the specific mode and features of the TLA® coaching method had not been used before within study coaches’ practice.

### Data analysis process

Data from all questionnaires received from participants at all time points was analysed for comparison of variables and analysis undertaken (for nominal data) and correlation for possible relationships and emerging themes. Data was analysed descriptively to summarise the information from each of the questionnaires, providing median scores where appropriate. Free-text responses were also recorded from returned questionnaires as well as pertinent information that was elicited through semi-structured interviews at time point T4 with participants and coaches which made use of a topic guide created by the researcher. Interviews took place 1 month after each participant completed their study arm and focused on the experience in undertaking reflexive action learning processes (participants) and providing the specific AL intervention (coaches). All coaching sessions (*n* = 9) and interviews (*n* = 6) were audio-taped, and then transcribed verbatim by the researcher. The transcripts were analysed using thematic analysis to support creation of themes [18] and exploration of possible points of commonality, similarities and/or differences between participants.

## Results

Results are shown below divided into quantitative and qualitative results collected and analysed at each time point T0 to T4.

### Time point T0 (pre-post) – Quantitative

**Fig. 4:**
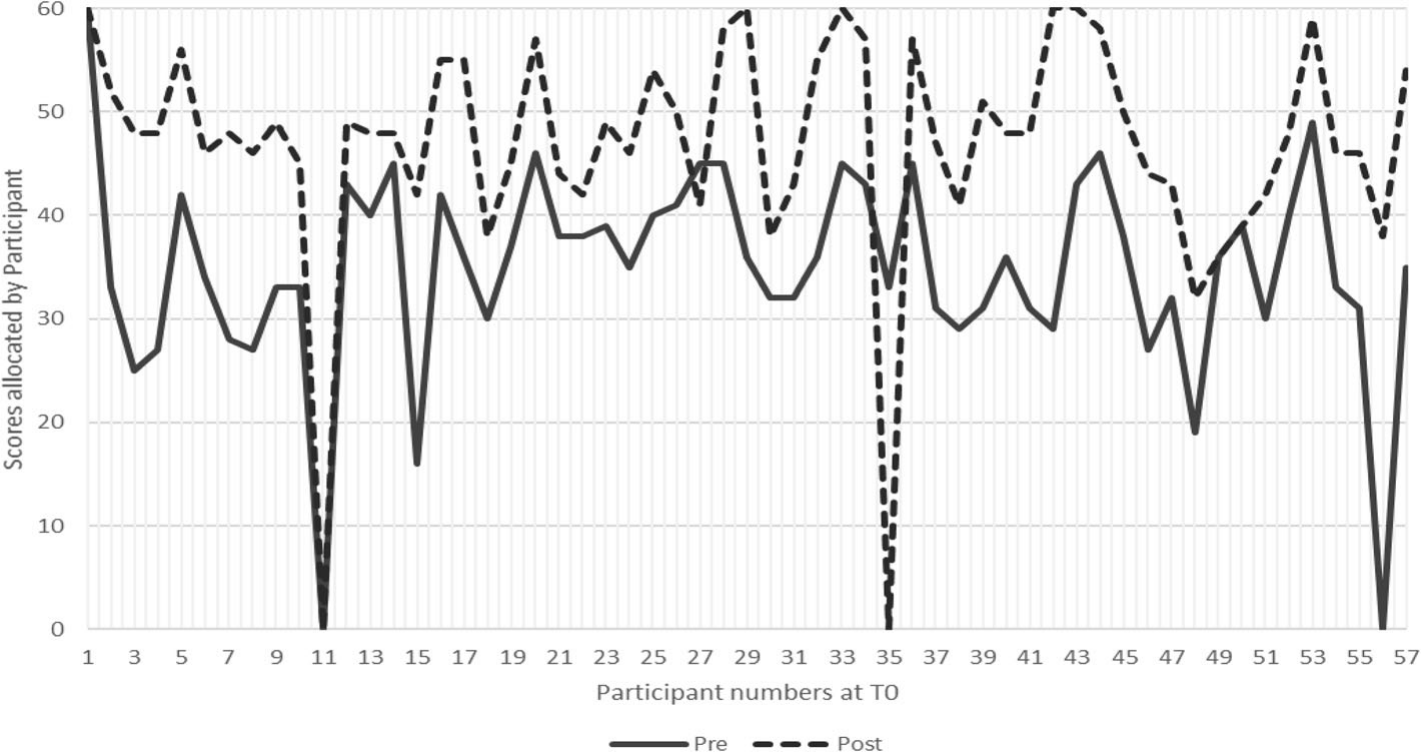
Baseline scores at T0 showing pre-post perceptions

Scoring undertaken at baseline (T0) from the full dataset of 57 learners demonstrate effectiveness of training, measuring at an average of 35% improvement in perceived levels of knowledge, skills, and confidence, as shown by Fig. 4. Scores ranged from 16 to 60 (maximum score) at pre-course data collection and 32–60 at immediate post-course. The overall trend shows that 92% of learners measured a perceived positive change (improvement) in score from pre- to post-course. 6% of learners perceived no difference in knowledge, skills and confidence, and 2% deemed learning had not improved following training.

### Time point T0 (pre-post) qualitative

From the questionnaires, learners were asked to outline anything that they felt they had learnt which they would do differently in practice, having reflected on their current levels and future planned use of their knowledge, skills, and confidence. The following table (Table 1) shows themes arising from, and supported by, evidence from free text data returned by learners:

**Table 1:** Themes arising from learner perception immediate post-course time point (T0)

Themes at T0	Evidence from learner data to support creation of themes:
Increase/improvement in specific KSC from course experience and individual learner needs, specifically: • Silence • Dealing with difficult questions • Breaking bad news • Listening • Use of cues/information gathering	Elicit information and use my empathy to build trust (Nurse) I’m more aware of that gathering of information, listening for that cue, since the workshop. It’s unlocked a few things I had in my artillery, helped me move to the next level (ANP) Reflections, using or avoiding certain phrases (Consultant) My objective was to explore silence – it’s a powerful tool to develop relationships with patients and to see how it can transform relationships was powerful for me. By using silence, we allow the patient to consider in their own time, it’s no longer my agenda (GP)
Immediate and planned improvements in professional practice	Not focus on practical outcomes but focus on feelings (NPOP) Will always make plan before any situation, (course) will enable me to be adequately equipped for EoLC discussions (DN) The course reminded me of the importance of decisions based on good communication – it’s been a way for me to highlight and improve the care in the organisation where I work as I can facilitate these discussions (GP) (Course) will definitely help guide how I engage with patients, carers, and other professionals (CNS)
Reflexivity	Role play has allowed me to re-assess how I deliver information, now more confident in tackling sensitive subjects (Nurse) Use of silence, blocking behaviours and the Calman Gap made me assess patients differently, this will enhance my practice. To be less fearful of exploring patient emotions and feelings (CNS) It was a safe space to practice and make mistakes, to learn from others, get advice, tips, and support (GP)

These results provide evidence of significant perceived learning following training, in and of itself, but importantly, serve as a vital baseline measure for the research for scheduled follow-up (time points T1 to T3).

### Time point T1-T3 - quantitative

**Fig. 5:**
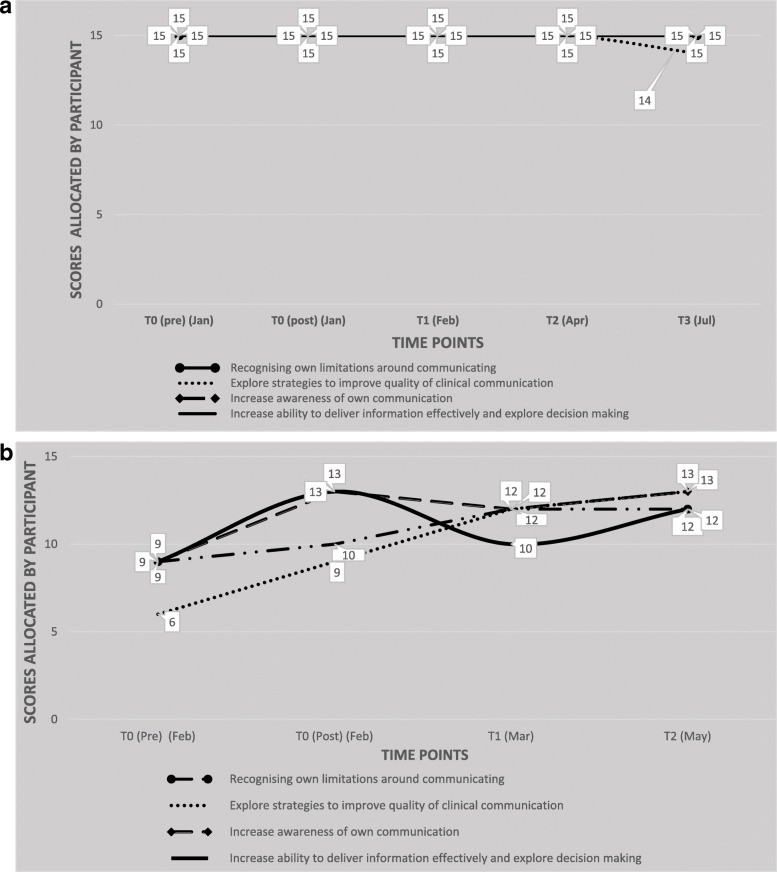
**a** Self-assessed scores from Control Group participant C1 broken down by Communication Skill area. **b** Self-assessed scores from Control Group participant C2 broken down by Communication Skill area

**Fig. 6:**
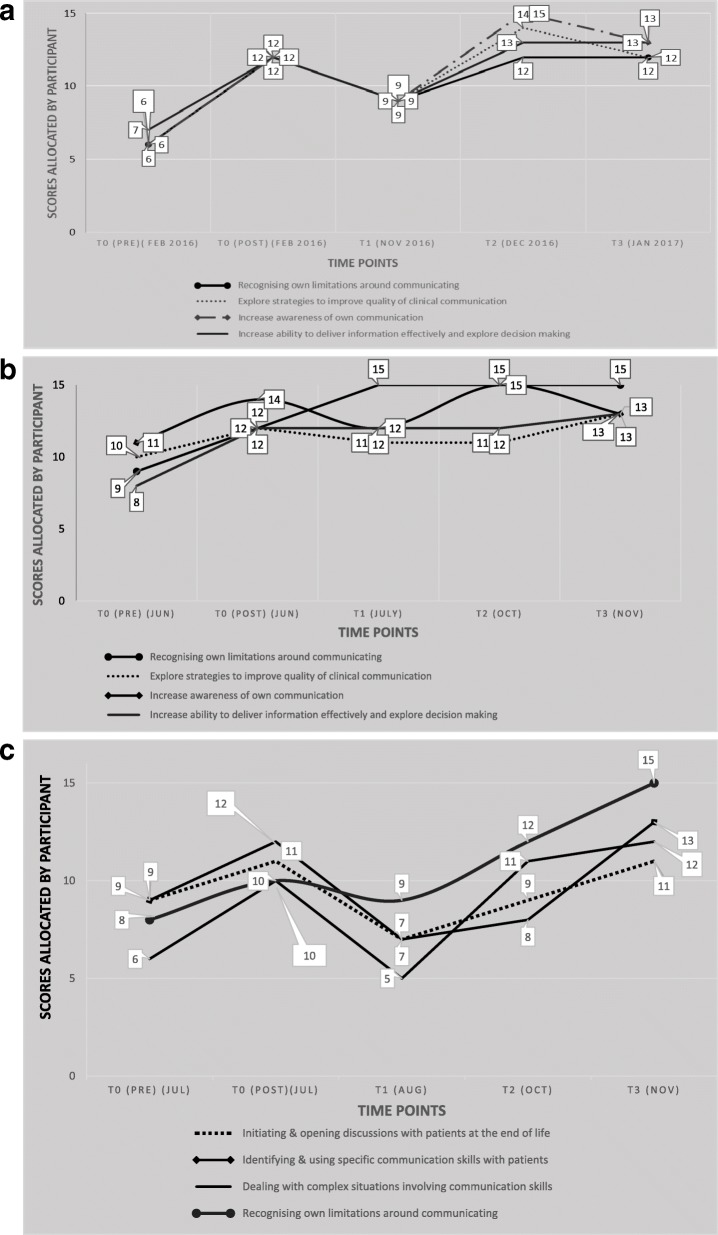
**a** Self-assessed scores over time for Intervention participant IN1 broken down by Communication Skill area. **b** Self-assessed scores over time for Intervention participant IN2 broken down by Communication Skill area. **c** Self-assessed scores over time for Intervention participant IN4 broken down by Communication Skill area

The overall average measure of perceived improvement was calculated at 46% based on participants returning questionnaires to time point T3. The Control arm’s combined improvement score measured at 25% and the Intervention arm’s at 67% improvement from pre-course to the ends of their respective learning journeys at T3. The following graphs (Fig. 5 a, b and Fig. 6 a, b, c) illustrate the learning journeys as experienced and reported by each completed participant and are shown by study arm. The intention for all participants was to return the questionnaires at T1 (1 month after training), T2 (2 months after training) and T3 (3 months after training). As demonstrated by the individual learning journeys, timely returns of the questionnaire were variable and whilst some participants adhered to the requested time points, some did not. This is indicated in each graph.

### Control arm

Despite being assumed “lost to study” through non-return of data at time point T3, the learning journey experienced and reported by participant C2 nevertheless warrants inclusion, not least to show the contrast with participant C1. For the participants within this study arm, an average measure of 25% perceived improvement in levels of knowledge, skills and confidence was reported. Although this data suggests a lower than average overall improvement, the learning journey for these individuals is nevertheless clear and qualitative data at time point T4 supports the value of the approach by the participants themselves.

### Intervention arm

The average measure of perceived improvement for completed participants in the Intervention arm was calculated at 67% from T0 to T3, demonstrating a significant perceived increase in all areas of knowledge, skills and confidence. This is further evidenced by reflexive comments within the questionnaire returns as well as through final interviews with all completed participants (*n* = 4) and coaches providing intervention (*n* = 2) as per Table 2.

**Table 2:** Themes arising from participation and experience in action learning (T4)

Themes at T4	Evidence in support of theme
Experience of follow-up processes and activity	Process was useful, good opportunity to reflect (CONTROL PARTICIPANT) Helpful framework for reflections and action planning (CONTROL PARTICIPANT) Having the opportunity to come back (after a course) and revisit, highlight what’s important has been useful (INTERVENTION PARTICIPANT) It was difficult and at times, challenging, coaching someone I’d never met or seen, I tend to have a visual mind (COACH) Having an action plan, a clear structure and clear outcomes really helped focus the sessions (COACH) I’ve really enjoyed the telephone coaching sessions, on one level it helps because listening skills really come into their own, and you’re not distracted by observing, or getting into personal realms (COACH)
Time difficulties and challenges of continuing with the research	The reality is, time is always the issue (CONTROL PARTICIPANT) Flexibility is important and the ability to do that outside of regular hours (INTERVENTION PARTICIPANT) It was quite difficult to commit to but every time I got to a coaching session or sit down and do my action plan, I found it quite useful, so it was swings and roundabouts (INTERVENTION PARTICIPANT) Despite logistics of scheduling and availability, it didn’t have a negative impact on the experience of the intervention (COACH) I think the 30 min only rule may be a discipline for the coach but I wouldn’t criticise it as it is sometimes what the participant needs (COACH)
Use of coaching as a tool for development	Coaching has been a useful mechanism to reflect verbally, I needed to hear myself talk about these things, it reminds me why I’m doing them (INTERVENTION PARTICIPANT) I loved the coaching. I don’t really like questionnaires, but that sort of human interaction, having somebody else helping you structure your ideas, has been really effective for me (INTERVENTION PARTICIPANT) I think definitely using coaching after your education events just reinforces your learning (INTERVENTION PARTICIPANT) It helped to tackle things straight on rather than avoid them, so I spent time discussing things with [coach], things I specifically wanted to do (INTERVENTION PARTICIPANT) It’s something that should be instilled as part of our foundation rather than ad hoc when we hit problems (INTERVENTION PARTICIPANT) 30 min is a really short period of time, but if you’re focused on a very specific topic, it’s probably about right (COACH) I think it’s a really powerful additional training intervention (COACH) Even with missed sessions and cancellations, there is a lot of value and feasibility in this kind of approach (COACH) The action learning via coaching model is helpful and adds value and I can see how it can support different learning styles and levels (COACH)
Impact of training on communicating with patients on staff	Sometimes I feel guilty about just sitting down and talking to patients…if you’re not seen rushing around when everyone else is busy I feel guilty. It’s an entrenched professional dilemma (INTERVENTION PARTICIPANT) My contact with patients is more natural and the dynamic is very different. I used to feel unsettled and now I don’t. I’m comfortable sitting in silence (INTERVENTION PARTICIPANT) I’ve been taking time to listen more, create more time for the patient or family, but it has an impact on the way I feel, you know the guilt of somebody else picking the pieces whilst you sit with a patient for 20 min instead of just overviewing (INTERVENTION PARTICIPANT)
Responsibility for own learning and development	(Using this research project) I thought it might provide me with a bit of support, be a positive focus to keep what I’ve learnt close and not to let it slip by for a while, until it builds into my everyday practice (INTERVENTION PARTICIPANT) It prompted me to do some sort of further learning on my own back (INTERVENTION PARTICIPANT) I’m going to incorporate action planning into my goals, I will put it in my PPRD and update my goals (INTERVENTION PARTICIPANT) I need to review, rewrite, and reflect on my goals so I can use the different frameworks from the course as well as capture what I’ve learnt and how I’ve changed (INTERVENTION PARTICIPANT) I could see how the coaching triggered different, latent thinking, which our conversations really seemed to bring to the surface (COACH)
Use of reflexivity as an action learning activity	Have never been asked to reflect on my thoughts after a communication skills training, it was first of its kind. (It) made me come away and think about what else I could do to introduce into my professional practice and I will follow that up (CONTROL PARTICIPANT). It really allowed me to revisit some of the previous things I’d been thinking, but in a structured way (INTERVENTION PARTICIPANT) It wasn’t until you’ve done it, do you realise the full effectiveness of it (INTERVENTION PARTICIPANT) In hindsight, you don’t really realise until you’re analysing, what you do and how you do things (INTERVENTION PARTICIPANT) I’m reflecting back and thinking if I hadn’t gone on that course when I did, I may not have had that conversation I needed to have with that patient, and I would have been sitting here thinking, it’s too late now. I was really happy I’d addressed that (difficult) situation (INTERVENTION PARTICIPANT) I feel that my self-awareness and my understanding and skills have definitely improved since working on them and I’ve been examining and reflecting on my own behaviours (INTERVENTION PARTICIPANT) I came into the first session with a list of three things I wanted to work on and by the end of that session they had changed, so in my next action plan I had that updated to reflect what I actually wanted to work on (INTERVENTION PARTICIPANT) My thinking has changed, my working style has changed, I’ve had time to reflect on things, gauge where I’m going (INTERVENTION PARTICIPANT) The model is a different way of approaching AL and reflexivity in a much stronger way (COACH)

### Time point T4 – Qualitative

Rich data was collected via semi-structured interviews at time point T4, and brought together with qualitative data collected from questionnaires at time points T0-T3. This was analysed inductively and supported the creation of several important themes, with respect to the experience of action learning processes and activity, use of reflexivity within and outside of coaching, and the responsibility for learning and development, even with the accepted challenges and constraints of time and logistics. These were then grouped by theme as can be seen from Table 2.

Through the qualitative data gathered and analysed from the questionnaires and interviews, using Creswell’s [17] framework, it can also be seen that that the application and process of action learning and reflexivity during all time-points was considered useful for all study arm participants. This provided a foundation for the perceived utility and feasibility of action learning processes and method in facilitating effective learning transfer. In some instances, this has had a catalytic effect for motivation for immediate and planned improvements in professional practice, as well as generating ideas for ongoing improvement and planning for longer-term team benefit.

### Combining the data

It is important to combine the results of both quantitative and qualitative data at time points from T0-T3 to understand the current state of play for a particular cohort of participants, or an individual, at specific points in time. Participants were given the opportunity to reflect on the process of follow-up as well as their experience of it, thus enabling a deeper level of understanding for their own self-development, as well as acting as a triangulation [18] method for the research study. Therefore, in consideration of the combined data, the main outcome of the research demonstrates that, even with small numbers, there is clear evidence of higher perceived and reported levels of knowledge, skills and confidence for the majority of participants. Inferences may be drawn between the relationship between perceived levels of knowledge, skills and confidence for those who undertook the intervention and those that did not, accounting for any bias and restrictions or limitations found during the course of the study.

## Discussion

The general improvement from baseline to completion for Intervention participation (that was calculated from 35 to 67%), reinforces the idea that individuals within communities of practice can facilitate and strengthen collective learning [19]. Importantly, qualitative data from participants demonstrates that data collection processes and methods, whether intervention or control arm, provided learners with conducive conditions and a safe place to face anxieties of real-world problem solving, enabling reflection and subsequent personal and professional development [20–22].

Each participant, whether in the Control or Intervention arm of the study, used his or her own lived experience [23] applying interpretative analysis [24] to improve individual professional competency [25].Through the process of action learning and participatory practice-based research [26], individual knowledge and know-how was able to be expressed, perceptions and assessments of “self” and “organisation” were legitimised, ensuring that action planning for improvement was fully contextualised [27, 28]. This facilitates and contributes to bridging the gap between knowing *what* and knowing *how* to convert theory into practice [29]. The combination of *iterative process* and *action* of transferring knowledge into practice is a prime example of the SECI [30] model in action which addresses the needs of both individual and organisation in terms of improving knowledge, skills, and confidence to deliver an important clinical service at perhaps one of the most challenging times of life.

### Relevance to organisational learning

The results of the study also suggest organisational learning may be strengthened by the notion of transformational, intra-, and inter-organisational learning, which resonates with learners and those designing learning for meaningful and demonstrable change. Thus, action learning can be perceived to have utility as both a *method* and a *process* for healthcare professionals, their line managers, service-design professionals and ultimately service-users. Successful transformative learning from effective communication skills can only occur within organisations and across systems by bringing together the needs and expectations of these diverse stakeholders [26, 31].

### Utility and impact of measures and approaches

Learning from exploration and innovation is most likely to take place within a culture that values knowledge and innovation [32] and necessarily involves the discomfort of experiencing uncertainties, risks and ambiguities associated with iterative processes of potential change [33]. The type of learning occurring within the research is indicative of the double-loop learning [34] that utilises reflection on the learning process itself. This is evidenced by participants consistently describing their satisfaction with the process and its feasibility in seeking to determine insight. This indicates a shift from explicit knowledge to tacit and implicit knowledge [35, 36]. This is the point at which learning becomes “transformational” [37, 38] as underlying patterns of thinking change shape and lead to an informed change in behaviour, as evidenced by perception from some participants. When transformational learning occurs from individuals to their environments, this can act as a catalyst for the direction of knowledge to change, and its outcomes become more securely integrated into existing and new knowledge sets within specialised communities of professional practice [39]. Using knowledge and experience of participants from their own professional clinical practice as a foundation, a collective competence in communication skills may have formed the basis of a professional identity with specific insights found from the data [40].

### The importance of coaching within action learning and reflexivity

It has been said that coaching is informed by, and utilises a set of psycho-dynamic, goal-seeking and solution-focused principles [41–43] and that the coaching relationship is “one in which coach and coachee form a collaborative working alliance, articulate goals and develop specific action steps designed to facilitate goal attainment.” [42] Findings from this study arising from telephone-based coaching sessions are evidence of a strong behavioural solution-focused approach, with participants planning and executing actions, reflecting, and viewing success and continuing to evaluate next steps in the execution of professional duties. The ability to engage in constructive-style thinking of solutions is of particular importance when working in organisations that are in a state of flux and change [44]. Although the focus was not strictly connected with resilience, dealing with setbacks, and feeling an increased sense of personal mastery as challenges began to be overcome. This appeared to be an additional value-added benefit of coaching, which can also help to reduce general anxiety and stress, as seen in feedback from Intervention participants [45, 46]. Additionally, data from coaches’ perspectives triangulates well with participants’ perceptions, with coaches agreeing that, although variable, participants generally demonstrated a positive attitude to receiving coaching and held its value in high esteem. This is despite logistical challenges with diary availability and the relatively short time space for delivery (30 min). Even with differences in personal coaching styles and the experience in providing the intervention to participants, there is a positive appetite for the utility and feasibility of this type of action learning approach.

### Limitations of the study

There is often a risk that participants do not report behaviour fully or accurately [47, 48] and that participants from this sample population could easily demonstrate natural tendencies to “problem solve” rather than “learn though the process.” [49] Equally, the purpose and perceived benefits to participants in using this research to strengthen individual performance, calibre, and visibility [50] may have been intentional for those who consented to the study. Although 100% response rate can be illustrated at baseline (T0) and at the beginning of T1, limited numbers completed the study; in the Control group, 1 completed all three time points (T1-T3) and 1 withdrew after submitting data at T2, so final response rate was 16.67%. In Intervention Group, 3 completed all time-points, T1, T2, T3 (50% response rate). Following intervention, at T4, 4 participants remained, and were interviewed, thus overall 33% response rate for participants was recorded. Although coaches were also interviewed for their perceptions, these are not included as “participants.”

Given the lack of time and conflict with professional duties, agreeing to participate in this study may have compromised reflexive effort for some participants, and this may have been part of the reason for the numbers withdrawing or “lost to study” but without further investigation, this assumption cannot be proven. Other factors may have also contributed to the success (or otherwise) of levels and nature of completion rates. For example, the research study may have appealed to the personality and preferred learning styles of participants [5]. Participants with a more reflexive preference may have been more motivated to join a study offering these opportunities, or those without may have been deterred from engaging in such activities. Although the research was conducted according to ethical standards, utilising a consistent approach to data collection methods and processes, it is difficult to establish if non-verbal communication encouraged a skewed response to demonstrate a favourable response to the research in question. Without independently assessing researcher and/or participant bias, reliability and validity [51] cannot therefore be fully established.

## Conclusions

The study has demonstrated the notion of value and benefits of action learning as perceived by participants, triangulated and supported by the experience of those providing the intervention. Although more work is required on closing the gap between theory and practice [52] and between designing, measuring and linking behavioural outcomes, action learning can offer an innovative alternative to traditional forms of learning for sustainable change [53]. Although numbers were limited, a positive experience reported by participants in both study arms provides assurance that action learning is feasible within this community of practice and could support effective learning transfer.

## Abbreviations

ACST: Advanced Communication Skills Training
AL: Action Learning
ANP: Advanced Nurse Practitioner;
CCST: Core Communication Skills Training
CMPEoLCN: Cheshire & Merseyside Palliative & End of Life Care Network
CNS: Clinical Nurse Specialist
DN: District Nurse
GP: General Practitioner
ICST: Intermediate Communication Skills Training
KSC: Knowledge, Skills, Confidence
NPOP: Nurse Practitioner for Older People
PPRD: Personal Professional Review & Development
SECI: Socialisation, Externalisation, Combination, Internalisation model
T0: Time point 0
T1: Time point 1
T2: Time point 2
T3: Time point 3
T4: Time point 4
